# Comparison of eight modern preoperative scoring systems for survival prediction in patients with extremity metastasis

**DOI:** 10.1002/cam4.6097

**Published:** 2023-06-12

**Authors:** Tse‐Ying Lee, Yu‐An Chen, Olivier Q. Groot, Hung‐Kuan Yen, Bas J. J. Bindels, Robert‐Jan Pierik, Hsiang‐Chieh Hsieh, Aditya V. Karhade, Ting‐En Tseng, Yi‐Hsiang Lai, Jing‐Jen Yang, Chia‐Che Lee, Ming‐Hsiao Hu, Jorrit‐Jan Verlaan, Joseph H. Schwab, Rong‐Sen Yang, Wei‐Hsin Lin

**Affiliations:** ^1^ Department of Orthopaedic Surgery National Taiwan University Hospital Taipei Taiwan; ^2^ Department of Medical Education National Taiwan University Hospital Taipei Taiwan; ^3^ Department of Orthopaedic Surgery University Medical Center Utrecht–Utrecht University Utrecht Netherlands; ^4^ Department of Orthopaedic Surgery Massachusetts General Hospital–Harvard Medical School Boston USA; ^5^ Department of Orthopaedic Surgery National Taiwan University Hospital Hsin‐Chu Taiwan; ^6^ Department of Medical Education National Taiwan University Hospital Hsin‐Chu Taiwan

**Keywords:** Asian cohort, external validation, extremity metastasis, survival prediction models

## Abstract

**Background:**

Survival is an important factor to consider when clinicians make treatment decisions for patients with skeletal metastasis. Several preoperative scoring systems (PSSs) have been developed to aid in survival prediction. Although we previously validated the Skeletal Oncology Research Group Machine‐learning Algorithm (SORG‐MLA) in Taiwanese patients of Han Chinese descent, the performance of other existing PSSs remains largely unknown outside their respective development cohorts. We aim to determine which PSS performs best in this unique population and provide a direct comparison between these models.

**Methods:**

We retrospectively included 356 patients undergoing surgical treatment for extremity metastasis at a tertiary center in Taiwan to validate and compare eight PSSs. Discrimination (c‐index), decision curve (DCA), calibration (ratio of observed:expected survivors), and overall performance (Brier score) analyses were conducted to evaluate these models’ performance in our cohort.

**Results:**

The discriminatory ability of all PSSs declined in our Taiwanese cohort compared with their Western validations. SORG‐MLA is the only PSS that still demonstrated excellent discrimination (c‐indexes>0.8) in our patients. SORG‐MLA also brought the most net benefit across a wide range of risk probabilities on DCA with its 3‐month and 12‐month survival predictions.

**Conclusions:**

Clinicians should consider potential ethnogeographic variations of a PSS's performance when applying it onto their specific patient populations. Further international validation studies are needed to ensure that existing PSSs are generalizable and can be integrated into the shared treatment decision‐making process. As cancer treatment keeps advancing, researchers developing a new prediction model or refining an existing one could potentially improve their algorithm's performance by using data gathered from more recent patients that are reflective of the current state of cancer care.

## INTRODUCTION

Metastatic lesions in the extremities can lead to pathologic fractures, pain, immobility, and compromised quality of life.^1^, ^2^, ^3^, ^4^ Although surgical intervention can often relieve symptoms and prevent some pathologic fractures, it is important to weigh the benefits against the risks associated with surgery in patients with bone metastasis because they tend to have a limited lifespan. Patients with a very short life expectancy might be better managed with non‐surgical treatment (e.g., radiotherapy) or minimally/less invasive procedures for symptomatic relief; Patients expected to have a longer survival, on the other hand, could benefit from more extensive surgery such as tumor resection and prosthetic replacement to prevent local recurrence and reconstruction failure.^5^, ^6^ In 2020, the Musculoskeletal Tumor Society (MSTS), American Society for Radiation Oncology (ASTRO), and American Society of Clinical Oncology (ASCO) jointly prepared a clinical practice guideline for the treatment of metastatic carcinoma and myeloma of the femur, in which the expert panel suggested surgeons “utilize a validated method of estimating survival of the patient in choosing the method of reconstruction”.^7^ Predicted survival is now recognized as an important factor that could influence decision‐making and procedure selection in the management of skeletal metastasis.

Survival estimation in patients with advanced cancer, however, is not easy. Several preoperative scoring systems (PSSs) that incorporate various clinical and demographic factors such as age, gender, primary tumor type, and laboratory values have been developed and validated in the past for this purpose using Western cohorts.^8^, ^9^, ^10^, ^11^, ^12^, ^13^, ^14^ However, evidence exists that Han Chinese people with certain types of malignancies such as breast and prostate cancer had a better prognosis than their Western counterparts.^15^, ^16^, ^17^, ^18^ This raises the question whether PSSs developed in western countries can be readily applied onto patients in other ethnogeographic regions. In our previous work, we found that the Skeletal Oncology Research Group machine learning algorithm (SORG‐MLA) retained good performance in patients of Han Chinese descent. Yet little is known regarding the model performance of other existing PSSs in this ethnic population.^19^

In this study, we asked whether most western‐developed PSSs were generalizable to a Taiwanese cohort mainly composed of Han Chinese patients, and attempted to demonstrate how these PSSs performed in a head‐to‐head comparison.

## MATERIAL AND METHODS

### Study design

This study was designed following the transparent reporting of a multivariable prediction model for individual prognosis or diagnosis (TRIPOD)^20^, ^21^ and the preferred reporting items for systematic reviews and meta‐analyses (PRISMA)^22^ guidelines. It was approved by our research ethics committee (201912022RIND) and registered online at PROSPERO (CRD42021266033).

### Search strategy

**FIGURE 1:**
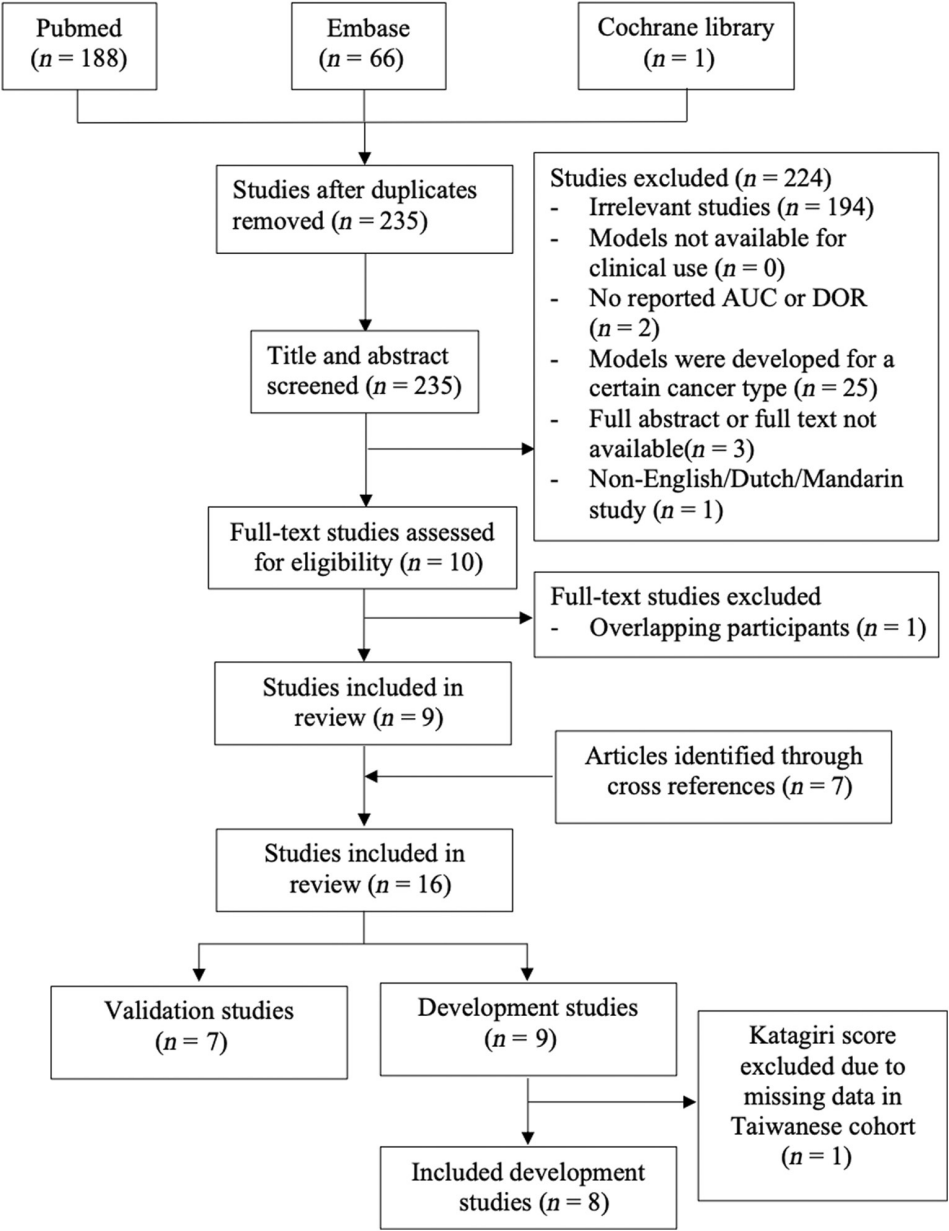
Flowchart of included studies. DOR = diagnostic odds ratio; AUC = area under curve.

PubMed, Embase, and Cochrane were searched as of December 20, 2021 with no time constraint. The following keywords and their respective combinations were used: “survival”, “prediction model”, and “extremity metastases” (Table S1). Articles that cited the developmental studies were also screened (Figure 1).^8^, ^9^, ^10^, ^11^, ^12^, ^13^

The inclusion criteria for studies were: (1) full text; (2) developing and/or validating a PSS of survival prediction for patients with extremity metastases; and (3) providing web‐based applications or formulas of the PSS for clinical use. The exclusion criteria for studies were: (1) not written in English; (2) developing and/or validating models designed for a specific tumor type or sarcoma; (3) developing and/or validating models designed only for spinal metastases; and (4) not reporting concordance index (c‐index) or odds ratio for discrimination analysis. Two authors independently screened all studies using the predefined criteria and assessed methodological quality. Disagreements were resolved by discussion with a third author.

### Study characteristics

**FIGURE 2:**
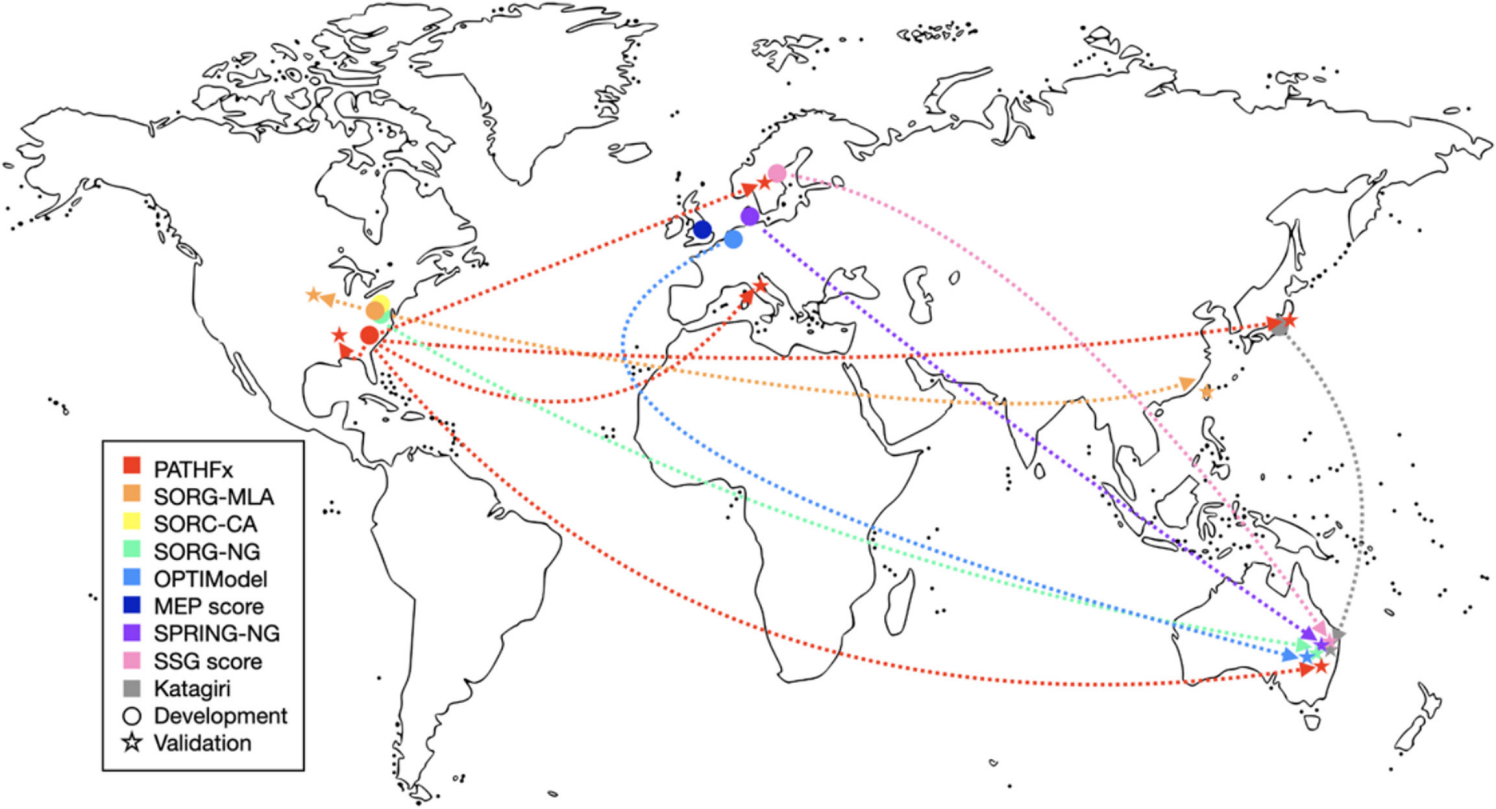
World map of the development and validation studies.

In total, 16 studies were included consisting of nine development and seven validation studies (Figures 1 and S1). The nine PSSs included: PATHFx,^11^, ^12^, ^23^, ^24^ Scandinavian Sarcoma Group (SSG),^8^ SORG classical algorithm (SORG‐CA), SORG nomogram (SORG‐NG),^25^ SPRING nomogram (SPRING‐NG),^9^, ^13^ OPTIModel,^26^ Metastatic Early Prognostic (MEP) score,^10^ SORG‐MLA,^27^ and revised Katagiri score^28^, ^29^ (Table 1). The revised Katagiri score was not validated in this cohort due to missing data of c‐reactive protein (CRP) and lactate dehydrogenase (LDH), leaving eight PSSs for validation (Figure 2).

**TABLE 1:** Summary and comparison of included studies and our study.

Author	Study characteristics						Patient characteristics			
	Year	Country	Study period	Institution/Cohort	Proportion of Hanzu^b^	Type of studies	Sample size	Endpoints	Mortality; *n* (%)	AUC (95%CI)
PATHFx										
Forsberg	2011	USA	1999–2003	MSKCC	1.5%	Development	189	3 months	60 (32%)	0.85 (0.80–0.93)
								12 months	110 (58%)	0.83 (0.77–0.90)
Ashley^a^	2019	USA	2012‐2016	MDR	1.5%	Validation	192	1 month	6 (3%)	0.82 (0.68–0.95)
								3 months	35 (18%)	0.83 (0.77–0.90)
								6 months	65 (34%)	0.79 (0.73–0.86)
								12 months	105 (55%)	0.79 (0.73–0.86)
								18 months	129 (67%)	0.79 (0.72–0.86)
								24 months	137 (71%)	0.76 (0.69–0.84)
Ashley^a^	2019	USA	2016–2018	IBMR	1.5%	Validation	197	1 month	17 (7%)	0.70 (0.58–0.82)
								3 months	71 (36%)	0.77 (0.70–0.84)
								6 months	102 (52%)	0.77 (0.70–0.83)
								12 months	129 (66%)	0.78 (0.71–0.85)
								18 months	151 (77%)	0.79 (0.71–0.86)
								24 months	160 (81%)	0.82 (0.75–0.90)
Forsberg	2012	Scandinavia	1999–2009	SSMR	0.4%	Validation	815	3 months	258 (32%)	0.79 (0.76–0.82)
								12 months	574 (70%)	0.76 (0.72–0.80)
Piccioli	2015	Italy	2010–2013	OORC	0.5%	Validation	287	3 months	20 (7%)	0.80 (0.72–0.88)
								12 months	106 (37%)	0.77 (0.72–0.82)
Ogura	2017	Japan	2009–2015	NCCH, CIH, UTH, TUH, JUH	0.6%	Validation	261	1 month	21 (8%)	0.77 (0.63–0.86)
								3 months	43 (17%)	0.80 (0.72–0.87)
								6 months	82 (31%)	0.83 (0.77–0.89)
								12 months	109 (42%)	0.80 (0.75–0.86)
Meares	2019	Australia	2003–2014	RNC, JHH	5.6%	Validation	114	3 months	38 (33%)	0.70 (0.69–0.70)
								6 months	56 (49%)	0.70 (0.69–0.70)
								12 months	79 (69%)	0.71 (0.70–0.71)
								24 months	95 (83%)	0.75 (0.74–0.75)
Lee	2022	ROC	2014–2019	NTUH	98%	Validation	356	1 month	17 (5%)	0.71 (0.58–0.84)
								3 months	63 (18%)	0.66 (0.59–0.73)
								6 months	108 (32%)	0.65 (0.59–0.71)
								12 months	167 (51%)	0.69 (0.64–0.75)
								18 months	191 (60%)	0.68 (0.62–0.75)
								24 months	204 (68%)	0.67 (0.60–0.74)
Katagiri										
Katagiri	2014	Japan	2005–2008	–	0.6%	Development	808	6 months	347 (43%)	–
								12 months	517 (64%)	–
								18 months	622 (77%)	–
								24 months	679 (84%)	–
Meares	2019	Australia	2003–2014	RNC, JHH	5.6%	Validation	114	6 months	56 (49%)	0.63
								12 months	79 (69%)	0.67
								24 months	95 (83%)	0.69
SSG										
Ratasvuori	2013	Scandinavia	1999–2009	SSG	0.4%	Development	651	6 months	273 (42%)	–
								12 months	384 (59%)	–
Meares	2019	Australia	2003–2014	RNC, JHH	5.6%	Validation	114	3 months	38 (33%)	0.63
								6 months	56 (49%)	0.64
								12 months	79 (69%)	0.62
Lee	2022	ROC	2014–2019	NTUH	98%	Validation	356	1 month	17 (5%)	0.65 (0.54–0.77)
								3 months	63 (18%)	0.64 (0.57–0.70)
								6 months	108 (32%)	0.60 (0.44–0.66)
								12 months	167 (51%)	0.66 (0.61–0.70)
								18 months	191(60%)	0.67 (0.62–0.73)
								24 months	204 (68%)	0.67 (0.61–0.73)
SORG‐CA										
Janssen^a^	2015	USA	1999‐2013	MGH, BWH	1.5%	Development	927	1 month	–	0.67 (0.55–0.78)
								3 months	250 (27%)	0.70 (0.62–0.77)
								12 months	538 (58%)	0.68 (0.61–0.75)
Lee	2022	ROC	2014–2019	NTUH	98%	Validation	356	1 month	17 (5%)	0.59 (0.47–0.72)
								3 months	63 (18%)	0.63 (0.56–0.70)
								12 months	167 (51%)	0.67 (0.62–0.72)
SORG‐NG										
Janssen^a^	2015	USA	1999‐2013	MGH, BWH	1.5%	Development	927	1 month	–	0.72 (0.59–0.84)
								3 months	250 (27%)	0.75 (0.68–0.83)
								12 months	538 (58%)	0.73 (0.65–0.80)
Meares	2019	Australia	2003–2014	RNC, JHH	5.6%	Validation	114	3 months	38 (33%)	0.68
								12 months	79 (69%)	0.71 (0.70–0.71)
Lee	2022	ROC	2014–2019	NTUH	98%	Validation	356	1 month	17 (5%)	0.64 (0.48–0.79)
								3 months	63 (18%)	0.70 (0.63–0.77)
								12 months	167 (51%)	0.74 (0.69–0.80)
SORG‐MLA										
Thio	2020	USA	1999–2017	MGH, BWH	1.5%	Development	1090	3 months	305 (29%)	0.87 (0.86–0.88)
								12 months	639 (62%)	0.85 (0.83–0.86)
Tseng	2021	Taiwan	2014–2019	NTUH	98.0%	Validation	356	3 months	63 (18%)	0.80 (0.74–0.86)
								12 months	167 (51%)	0.84 (0.80–0.89)
Skalitzky	2021	USA	2003–2019	UIHC	1.5%	Validation	264	3 months	51 (19%)	0.83 (0.76–0.88)
								12 months	110 (42%)	0.84 (0.79–0.88)
Lee	2022	ROC	2014–2019	NTUH	98%	Validation	356	3 months	63(18%)	0.80 (0.74–0.86)
								12 months	167(51%)	0.84 (0.80–0.89)
OPTIModel										
Willeumier	2018	Netherlands	2000–2013	LUMC, UMGG, UMCU, HMC, EMC, RDGG	0.5%	Development	1150	1 month	99 (9%)	–
								3 months	349 (31%)	–
								6 months	535 (47%)	–
								12 months	722 (64%)	–
								24 months	900 (80%)	–
Willeumier	2018	Austria	2000–2013	MUG	0.2%	Validation	250	1 month	20 (10%)	–
								3 months	61 (29%)	–
								6 months	103 (49%)	–
								12 months	133 (64%)	–
								24 months	162 (77%)	–
Meares	2019	Australia	2003–2014	RNC, JHH	5.6%	Validation	114	3 months	38 (33%)	0.66
								6 months	56 (49%)	0.67
								12 months	79 (69%)	0.79 (0.78–0.79)
								24 months	95 (83%)	0.77 (0.77–0.77)
Lee	2022	ROC	2014–2019	NTUH	98%	Validation	356	1 month	17 (5%)	0.67 (0.54–0.80)
								3 months	63 (18%)	0.69 (0.63–0.76)
								6 months	108 (32%)	0.65 (0.59–0.71)
								12 months	167 (51%)	0.70 (0.64–0.75)
								18 months	191 (60%)	0.68 (0.63–0.74)
								24 months	204 (68%)	0.68 (0.62–0.74)
SPRING‐NG										
Sørensen	2016	Denmark	2003–2008	CUHR	0.3%	Development	130	3 months	43 (33%)	0.79 (0.66–0.90)
								6 months	66 (50%)	0.81 (0.70–0.91)
								12 months	81 (62%)	0.85 (0.74–0.94)
Sørensen	2018	Denmark	2003–2013	CUHR	0.3%	Validation	270	3 months	–	0.82
								6 months	–	0.83
								12 months	150 (56%)	–
Sørensen	2018	Denmark	2014–2016	CUHR	0.3%	Validation	164	3 months	–	0.82 (0.73–0.91)
								6 months	–	0.85 (0.76–0.93)
								12 months	97 (59%)	0.86 (0.77–0.95)
Meares	2019	Australia	2003–2014	RNC, JHH	5.6%	Validation	114	3 months	38 (33%)	0.66
								6 months	56 (49%)	0.68
								12 months	79 (69%)	0.76 (0.75–0.76)
Lee	2022	ROC	2014–2019	NTUH	98%	Validation	356	3 months	63 (18%)	0.70 (0.63–0.77)
								6 months	108 (32%)	0.72 (0.66–0.78)
								12 months	167 (51%)	0.77 (0.72–0.80)
MEP										
Downie^a^	2019	UK	2010‐2016	UK trauma center	0.7%	Development	195	1 month	39 (20%)	–
								3 months	89 (46%)	‐
								12 months	154 (79%)	–
Downie^a^	2019	UK	2017	UK trauma center	0.7%	Validation	36	3 months	‐	0.82
Lee	2022	ROC	2014–2019	NTUH	98%	Validation	356	1 month	17 (5%)	0.59 (0.45–0.72)
								3 months	63 (18%)	0.66 (0.58–0.73)
								12 months	167 (51%)	0.61 (0.56–0.67)

^a^ These two or three cohorts came from the same study.

^b^ The proportion of Han Chinese was not provided in any of the studies and were therefore based on demographic numbers.^1^, ^2^, ^3^, ^4^, ^5^, ^36^, ^43^

Abbreviations: AUC, area under the receiver operating characteristics curve; BWH, Brigham and Women's Hospital; CI, confidence interval; CIH, Cancer Institute Hospital; CUHR, University Hospital Rigshospitalet, University of Copenhagen, Copenhagen, Denmark; UK, United Kingdom; EMC, Erasmus Medical Center, Rotterdam, the Netherlands; HMC, Haaglanden Medical Centre, The Hague; IMBR, International Bone Metastasis Registry; JHH, John Hunter Hospital; JUH, Juntendo University Hospital; MDR, Military Health System Data Repository; MGH, Massachusetts General Hospital; MSKCC, Memorial Sloan‐Kettering Cancer Center; NCCH, National Cancer Center Hospital; NTUH, National Taiwan University Hospital; OORC, 13 orthopedic oncology referral centers; RDGG, Reinier de Graaf Gasthuis, Delft; MUG, Medical University of Graz; RNC, Royal Newcastle Centre; SSG, Scandinavia Sarcoma Group; SSMR, Scandinavian Skeletal Metastasis Registry; TUH, Teikyo University Hospital; UIHC, LUMC, Leiden University Medical Centre; UMCU, University Medical Centre Utrecht; UMGG, University Medical Centre Groningen; USA, Untied States of America; UTH, University of Tokyo Hospital.

### Methodological quality assessment

Methodological quality assessment was assessed by Prediction model Risk Of Bias Asessment Tool (PROBAST),^30^, ^31^ which determines risk of bias and applicability of prediction models in systematic reviews. PROBAST consists of 20 signaling questions across four domains: participant selection, predictors, outcome, and analysis. Each domain is rated “low,” “high,” or “unclear”. The ratings of the four domains result in an overall risk of bias judgment.

Twelve studies (75%) displayed an overall high risk of bias, primarily due to risk of bias in analysis domain. Most studies used small datasets with inadequate outcome numbers or omitted key performance measures, such as discrimination and calibration (Figure S2, Table S2).^32^

### External validation cohort

All 397 patients ≥18 years old who underwent surgery for extremity metastasis at a tertiary center in Taiwan between 2014 and 2019 were retrospectively included (Figure S3). The indications for surgery were: (1) presence of a pathologic fracture, or impending fracture deemed unlikely to resolve with non‐operative treatment alone; and (2) patients considered fit for surgery based on a multi‐disciplinary assessment made by medical oncologist, anesthesiologist, and orthopedic surgeon. The exclusion criteria were: (1) patients with a diagnosis of sarcoma bone metastasis,^33^, ^34^ and (2) patients whose first surgery for extremity metastasis was not performed at our institution (Figure S3). 356 patients were eventually entered into the analyses. This cohort was the same as the one reported in our prior publication to validate SORG‐MLA on Taiwanese patients.^19^

### Outcomes and prognostic variables

Survival endpoints were defined as the time between index surgery for extremity metastasis and death by any cause. Data for survival could be ascertained for 356 (100%) patients at 1‐month, 350 (98%) at 3‐months, 342 (96%) at 6‐months, 326 (92%) at 12‐months, 314 (88%) at 18‐months, and 302 (85%) at 24‐months. Follow‐up was censored at 2 years postoperatively or at patient's death.

The following predictors were manually extracted to compute survival predictions by each PSS: age; gender; body mass index (BMI; kg/m^2^); Charlson comorbidity besides metastatic cancer; previous systemic and local radiation therapy; presence of visceral, brain, or lymph node metastases; number of bone metastases; impending or completed pathologic fracture; Eastern Cooperative Oncology Group (ECOG) performance status; American Society of Anesthesiology classification; primary tumor; and 10 preoperative laboratory values.^8^, ^28^, ^29^, ^35^, ^36^, ^37^ The surgeons’ estimation of survival for PATHFx was omitted as they were not recorded. Authors of SORG's development study did not participate in data extraction or analysis. The same definitions used in the development studies were adopted for both survival outcomes and predictor variables.

### Baseline characteristics

Of the 356 patients, 98% were of Han Chinese descent based on their self‐identified ethnicity in the admission record, which was significantly higher than the western counterparts.^38^, ^39^, ^40^, ^41^, ^42^, ^43^, ^44^ The median age was 61 years (range 25–95) and 184 patients (52%) were female (Table S3). The median BMI was 23 kg/m^2^ (range 13–39) and 215 patients (60%) had additional Charlson comorbidities. 283 patients (79%) had an ECOG of 0–2 while 73 patients (21%) had 3–4. The most common primary tumor types were lung (33%), breast (16%), and hepatocellular (10%). Mortality rate was 5% (17/356) at 1 month; 18% (63/350) at 3 months; 32% (108/342) at 6 months; 51% (167/326) at 12 months; 61% (191/314) at 18 months; and 68% (204/302) at 24 months.

### Missing data

The missForest technique was used to impute missing values for: sodium (0.3%), absolute lymphocyte and neutrophil count (2.2%), calcium (2.2%), alkaline phosphatase (5.0%), albumin (7.0%), and blood urea nitrogen (25%).^45^ Sensitivity analysis was performed using a subset of patients without missing data (*n* = 245; Table S4).

### Assessment of model performance and statistical analysis

Discrimination (c‐index), calibration (ratio of observed: expected survivors), overall performance (Brier score), decision curve analysis (DCA) and model consistency analysis were used as performance metrics.^46^

A c‐index = 1 indicates perfect discrimination, while a c‐index = 0.5 is equivalent to random guessing. Calibration refers to the agreement between the predicted outcomes and the actual outcomes, with a perfect calibration curve having an intercept of 0 and a slope of 1. A positive intercept indicates the actual outcome is generally underestimated by the prediction model, and a negative intercept suggests the opposite (overestimation).^47^, ^48^, ^49^ We also used log(O:E), the logarithm scale of the ratio of observed (O) to expected (E) survivors, to assess calibration. A log(O:E) > 0 signals an underestimation; and a log(O:E) < 0 an overestimation.^49^, ^50^ Calibration analysis could not be done for four PSSs SSG, MEP, SORG‐NG, and OPTIModel because they provided an integer score instead of an estimated survival probability.^9^, ^11^, ^26^, ^27^ The Brier score captures both discrimination and calibration and represents the model's overall performance. It ranges from 0 (perfect prediction) to 1 (worst prediction). The null‐model Brier score, which is derived by assigning a default prediction equaling the prevalence of the outcome at a given timepoint to each patient, should serve as a benchmark with which a prediction model's Brier score is compared. If the prediction model's Brier score is lower than that of the null model, then the model is considered having good performance. The magnitude of difference between a model's Brier score and the null model's Brier score can be compared among PSSs. The PSS with the most significant reduction is deemed as the best performer.

The DCA was designed to assess the clinical utility of a prediction model.^51^ It plots the net benefit of making the surgical decision (e.g., operate or not to operate on a patient) based on the model's prediction across all possible risk probabilities in relation to the default strategies of operating on all or no patients. The user of the model can decide on an acceptable risk probability, and determine if offering surgery, based on the model's survival prediction, would do more good than harm by assessing the corresponding net benefit. In general, if the risk associated with a proposed treatment is low, such as percutaneously ablating a tumor on a medically fit patient, a lower risk probability can be chosen. In contrast, if the proposed treatment carries an inherently high risk, for example, performing extensive tumor resection and complex reconstruction on a cachexic patient with large tumor burden, a higher risk probability should be adopted. Readers may refer to articles published by Vickers et al.^51^ and Karhade et al.^48^ for more detailed discussion on the use and interpretation of DCA.

Several PSSs predict survival at different timepoints. Based on the law of attrition by time, a patient's survival probability at short‐term should be higher than that at a longer term. For example, if a patient's 3‐month survival probability is estimated at 90% and 18‐month survival probability at 20%, these two predictions are considered “reasonable”. On the other hand, if a patient's 6‐ and 12‐month survival probability were estimated at 30% and 40%, respectively, these predictions would be clearly against intuition and deemed “unreasonable”. We defined model consistency (MC) as the ratio of intuitively reasonable prediction pairs to all prediction pairs. A MC = 0 meant all prediction pairs were against intuition; A MC = 1 indicated all predictions were intuitively reasonable. Model consistency analysis was not done for four PSSs that did not provide estimated survival probabilities.^9^, ^11^, ^26^, ^27^

## RESULTS

Eight studies validated nine models in the literature, with the PATHFx and SORG‐MLA having been repeatedly tested.^11^, ^12^, ^19^, ^23^, ^24^, ^25^, ^26^, ^27^, ^28^, ^29^ These PSSs provided discrimination ranging from acceptable to excellent in external validation studies with the following c‐indexes: PATHFx (0.70–0.85); Katagiri score (0.63–0.69); SSG (0.62–0.64); SORG‐CA (0.67–0.70); SORG‐NG (0.68–0.75); SORG‐MLA (0.80–0.84); OPTImodel (0.66–0.79); SPRING‐NG (0.66–0.86); and MEP (0.82) (Table 1). We could not compare calibration and DCA between PSSs because many studies did not report these metrics.

The performance of the eight PSSs tested on our Taiwanese cohort varied (Table 2). Only SORG‐MLA and SPRING‐NG were able to achieve a “good” discriminatory ability (defined as having a c‐index ≥0.7) across all their prediction time points. In general, the other six PSSs demonstrated fair or close to good discrimination with their survival estimations. When comparing the discriminatory abilities of the eight PSSs at 3 and 12 months (two time points often considered clinically important for treatment decision‐making), we found SORG‐MLA demonstrated the best discrimination (c‐index = 0.80; 95%CI = 0.74–0.86; and c‐index = 0.84; 95%CI = 0.80–0.89); At these two timepoints, SPRING‐NG provided the second‐best discrimination (c‐indexes, 0.70 and 0.77; Table 2), while the c‐indexes of PATHFx, SORG‐CA, MEP, and SSG, were all <0.70.

**TABLE 2:** C‐indexes and Brier scores of PSSs at different time points in the Taiwanese validation cohort (*n* = 356).

Performance metrics	PATHFx	SORG‐MLA	SORG‐CA	SORG‐NG	OPTIModel	MEP	SPRING‐NG	SSG
c‐indexes								
1 month	0.71 (0.58–0.84)	–	0.59 (0.47–0.72)	0.64 (0.48–0.79)	0.67 (0.54–0.80)	0.59 (0.45–0.72)	–	0.65 (0.54–0.77)
3 months	0.66 (0.59–0.73)	0.80 (0.74–0.86)	0.63 (0.56–0.70)	0.70 (0.63–0.77)	0.69 (0.63–0.76)	0.66 (0.58–0.73)	0.70 (0.63–0.77)	0.64 (0.57–0.70)
6 months	0.65 (0.59–0.71)	–	–	–	0.65 (0.59–0.71)	–	0.72 (0.66–0.78)	0.60 (0.44–0.66)
12 months	0.69 (0.64–0.75)	0.84 (0.80–0.89)	0.67 (0.62–0.72)	0.74 (0.69–0.80)	0.70 (0.64–0.75)	0.61 (0.56–0.67)	0.77 (0.72–0.82)	0.66 (0.61–0.70)
18 months	0.68 (0.62–0.75)	–	–	–	0.68 (0.63–0.74)	–	–	0.67 (0.62–0.73)
24 months	0.67 (0.60–0.74)	–	–	–	0.68 (0.62–0.74)	–	–	0.67 (0.61–0.73)
Brier score^a^								
1 month	0.04 (0.05)	–	0.05 (0.05)	0.04 (0.05)	0.04 (0.05)	0.05 (0.05)	–	0.04 (0.05)
3 months	0.14 (0.16)	0.12 (0.16)	0.14 (0.16)	0.14 (0.16)	0.14 (0.16)	0.15 (0.16)	0.14 (0.16)	0.14 (0.16)
6 months	0.20 (0.22)	–	–	–	0.20 (0.22)	–	0.19 (0.22)	0.21 (0.22)
12 months	0.22 (0.25)	0.16 (0.25)	0.23 (0.25)	0.20 (0.25)	0.22 (0.25)	0.24 (0.25)	0.20 (0.25)	0.23 (0.25)
18 months	0.21 (0.24)	–	–	–	0.21 (0.24)	‐	–	0.21 (0.24)
24 months	0.19 (0.22)	–	–	–	0.20 (0.22)	–	–	0.20 (0.22)

*Note*: The c‐indexes are provided with 95% confidence intervals between parentheses.

^a^ The Brier score of the null model is presented in parentheses.

Abbreviations: C‐indexes, concordance index; MEP, metastatic early prognostic score; PSS, preoperative scoring system; SORG, Skeletal Oncology Research Group; SORG‐CA, SORG classical algorithm; SORG‐MLA, SORG machine learning algorithm; SORG‐NG, SORG nomogram; SPRING‐NG, SPRING nomogram; SSG, Scandinavian Sarcoma Group.

The eight PSSs all had a lower Brier score compared with that of the respective null model at their various prediction timepoints (Table 2). At 3 and 12 months, SORG‐MLA had the most pronounced improvement in Brier score [0.04 (0.16–0.12) and 0.09 (0.25 to 0.16), respectively] among all eight PSSs. Sensitivity analysis using a subset of patients without missing data showed similar results (Table S4).

Calibration analysis was performed for four PSSs: PATHFx, SORG‐MLA, SORG‐NG, and SPRING‐NG (Table 3 and Figure S4). These four PSSs all had positive calibration intercepts at their respective prediction time points, indicating their overall tendency to underestimate the actual survival of patients in our cohort. At 3 months, SORG‐MLA had the best calibration (Figure S4B), with an intercept of 0.78 and a slope of 0.74. At 12 months, there was not a clear calibration winner at first glance (Figure S4D). However, in terms of the 12‐month log(O:E), PATHFx (log(O:E) = 0.36; 95%CI = 0.07–0.65) and SORG‐NG (log(O:E) = 0.35; 95%CI = 0.07–0.64) were the better two PSSs, with SORG‐MLA trailing slightly behind (log(O:E) = 0.45; 95%CI = 0.13–0.77). SPRING‐NG fared worst with a 12‐month log(O:E) of 0.62 (95%CI = 0.25–1.00).

**TABLE 3:** Calibration intercepts and slopes of four PSSs at different time points in the Taiwanese validation cohort (*n* = 356).

	PATHFx	SORG‐MLA	SORG‐NG	SPRING‐NG
Calibration intercepts				
1 month	1.00 (0.50–1.51)	–	0.92 (0.39–1.44)	–
3 months	1.60 (1.31–1.90)	0.78 (0.46–1.10)	0.88 (0.60–1.17)	0.64 (0.31–0.96)
6 months	1.39 (1.13–1.64)	–	–	0.80 (0.52–1.08)
12 months	0.61 (0.36–0.85)	0.75 (0.49–1.00)	0.60 (0.36–0.84)	1.02 (0.74–1.29)
18 months	0.42 (0.16–0.68)	–	–	–
24 months	0.52 (0.24–0.80)	–	–	–
Calibration slopes				
1 month	0.75 (0.28–1.21)	–	0.67 (−0.04 to 1.38)	–
3 months	0.51 (0.25–0.76)	0.74 (0.53–0.96)	0.90 (0.53–1.27)	0.56 (0.32–0.80)
6 months	0.52 (0.29–0.75)	–	–	0.72 (0.66–0.77)
12 months	0.71 (0.48–0.94)	1.22 (0.95–1.49)	0.88 (0.60–1.16)	0.63 (0.47–0.80)
18 months	0.59 (0.38–0.79)	–	–	–
24 months	0.51 (0.31–0.71)	–	–	–
Log(O:E)				
1 month	0.08 (−0.14–0.30)	–	0.07 (−0.14 to 0.28)	–
3 months	0.54 (0.08–0.99)	0.19 (−0.11 to 0.5)	0.23 (−0.06 to 0.52)	0.55 (0.20–0.91)
6 months	0.67 (0.27–1.07)	–	–	0.61 (0.27–0.94)
12 months	0.36 (0.07–0.65)	0.45 (0.13–0.77)	0.35 (0.07–0.64)	0.62 (0.25–1.00)
18 months	0.28 (−0.02–0.57)	–	–	–
24 months	0.38 (0.06–0.70)	–	–	–

*Note*: The 95 confidence intervals are provided between parentheses. The calibration results for the other four PSSs were not provided because they provided an integer score instead of an estimated survival probability.

Abbreviations: Log(O:E), the logarithm of the ratio of the observed survived number to the expected survival number; PSS, preoperative scoring system; SORG, Skeletal Oncology Research Group; SORG‐MLA, SORG machine learning algorithm; SORG‐NG, SORG nomogram; SPRING‐NG, SPRING nomogram.

**FIGURE 3:**
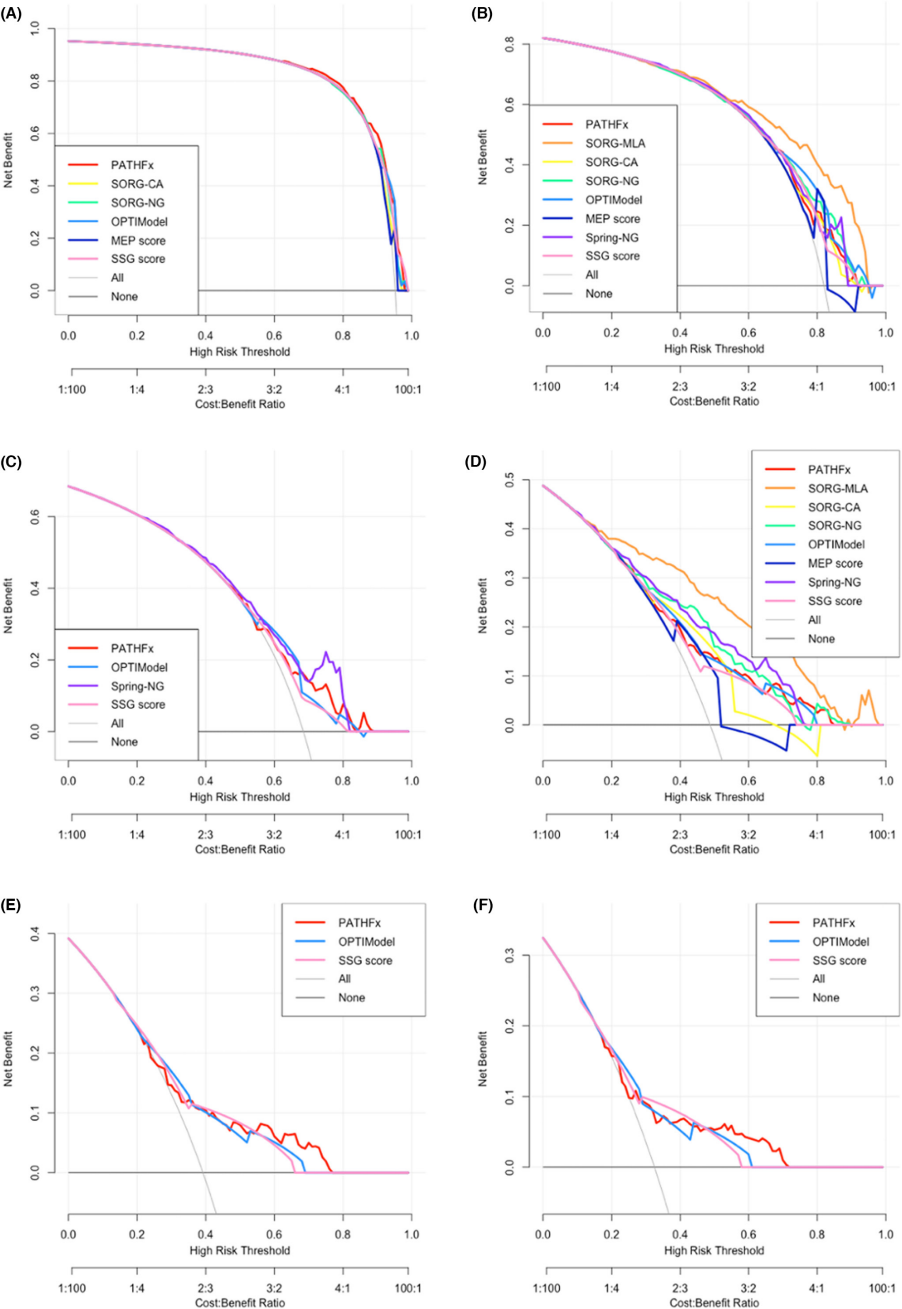
DCA plots of predictions by different PSSs are shown for (A) 1‐month; (B) 3‐month; (C) 6‐month; (D) 12‐month; (E) 18‐month; and 24‐month survival prediction. Color lines represent different PSSs: PATHFx (red); SORG‐MLA (orange); SORG‐CA (yellow); SORG‐NG (green); SPRING‐NG (violet); OPTIModel (light blue); MEP score (dark blue); and SSG score (pink).

On DCA, none of the eight PSSs provided significant benefits at 1 month. At 3 months, all PSSs provided net benefits when compared with a default strategy of operating on all or no patients (Figure 3B), and SORG‐MLA did so most distinctively and across the widest range of risk probabilities. At 12 months, the benefits of using PSSs are more pronounced (Figure 3D). Again, SORG‐MLA conferred the most pronounced clinical benefits across a wide range of risk thresholds.

SORG‐NG had a perfect MC of 1 for all its predictions. SORG‐MLA and SPRING‐NG were not far behind with MCs greater than 0.98 (Table S5). PATHFx occasionally exhibited subpar MCs (<0.9), such as when the 6‐month prediction was compared with the 12‐month, and when the 12‐month was compared with the 18‐month. For example, in our cohort there was an 81‐year‐old woman with breast cancer and multiple bone metastases, who had an ECOG scale of 3 before presenting with a pathologic femoral fracture. She had a hemoglobin of 10.7, an absolute lymphocyte count of 1.60, and no organ or lymph node metastasis on pre‐operatively workup. PATHFx estimated her chance of survival was 34% at 6 months and 37% at 12 months. Although one might argue these estimates were not necessarily clinically relevant, they did appear to be against the law of attrition by time.

## DISCUSSION

We identified in the literature nine PSSs developed in the past 30 years for survival estimation in patients with extremity metastases. As most PSSs were developed and validated using data from Western institutions, we sought to evaluate their generalizability in our predominantly Han Chinese population. In this study, SORG‐MLA had the best discrimination, overall performance, and brought the most net benefit on DCA at both 3 and 12 months. SORG‐MLA and SORG‐NG were the best calibrated at 3 and 12 months, respectively. While SORG‐NG, SORG‐MLA, and SPRING‐NG had perfect or almost perfect model consistencies, PATHFx occasionally produced counter‐intuitive predictions that resulted in suboptimal MCs. These findings suggest all PSSs do not perform equally on the same patient cohort. We believe most modern PSSs should be externally validated so that users are made aware of these models’ validity in populations other than the developmental cohort. Future researchers might consider developing algorithms using more contemporary and ethno‐geographically diverse data gathered through multi‐institutional and international collaboration to improve the generalizability of their prediction models.

There were several limitations in this study. First, 98% of the patients in our cohort are of Han Chinese descent. This uniformity of racial composition might decrease the referencing value of this study in populations with a low percentage of Han Chinese. Second, cancer treatment has a tremendous impact on patient survival but could vary by region and between healthcare systems. Physicians practicing in a clinical setting vastly different from ours may not find our findings completely applicable. Third, when interpreting performance of validation studies, one should be cognizant of potential publication bias and steer away from overzealous optimism because PSSs that perform poorly on external validation may be less often published.^52^ Fourth, MEP was designed only for patients with femoral metastasis but we validated it using patients with all types of extremity metastases, which might impact on its performance. However, we did not consider this a major limitation because the majority of our patients had femoral metastases and previous studies did not report significant survival differences among patients with metastases in different parts of the extremities.^53^ Lastly, we were unable to validate the revised Katagiri score because LDH and CRP were not routinely obtained in our institution. A previous validation study, however, demonstrated this scoring system provided only modest discriminatory ability.^54^ Despite these limitations, our study provides a comprehensive overview of several survival estimation tools in patients with extremity metastases, and validates them with a unique Han‐Chinese‐dominant cohort.

Most PSSs demonstrated a reasonable decline in discrimination in our Taiwanese cohort compared with their Western validations (Table 1 and 2). However, SORG‐MLA still achieved excellent discrimination at 3 and 12 months, with a c‐index of 0.80 and 0.84, respectively. Several reasons might contribute to SORG‐MLA's good performance. First, it was the newest algorithm and was created based on data from a large contemporary cohort that were more reflective of the current state of cancer treatment. This was a clear advantage of SORG‐MLA over the other PSSs because the relevance of clinical data toward future decisions decay over time.^55^ Second, SORG‐MLA took into account molecular factors that direct clinical treatment strategy, such as the mutational status of lung cancer and the hormonal receptor expression profile of breast cancer. Third, five state‐of‐the‐art machine‐learning techniques were tested during the development of SORG‐MLA, and only the best‐performing one (stochastic gradient boosting model) was selected and validated.^27^ This strategy, as opposed to training only one algorithm, could ensure that the model with the best performance was adopted. Fourth, SORG‐MLA requires more input variables (*n* = 15) than other PSSs (*n* = 7–9). In statistics, using more variables to predict an outcome will render better discriminatory results. Although in theory including more variables could also introduce the risk of overfitting and decrease the model's generalizability in newer, independent datasets, calibration analyses in this or other SORG‐MLA validation studies did not observe this issue.^19^, ^27^, ^56^ Our results indicated SORG‐MLA was a reliable survival prediction tool for Han Chinese patients with skeletal metastasis.

Some clinicians might not be well versed in statistical jargons like discrimination, calibration, and Brier score. A decision curve provides a visual representation of the value of using a certain PSS to estimate survival in the clinical setting. At 1 month, the eight PSSs in general provided little benefit across all risk thresholds. At 3 months, these PSSs offered a meaningful gain of benefit only if the risk of surgery exceeded 0.6, which was relatively high. At 12 months, however, most PSSs started to impart benefit when the risk of surgery was over 20%. These results suggest that PSSs may be especially useful when clinicians are considering if a patient with a short survival estimate, such as 3 months, should be offered more extensive surgery to address a problematic local bone metastasis. For patients with a longer survival such as 12 months, PSSs are beneficial when the proposed operation carries moderate to high risks.

We used model consistency (MC) to evaluate a PSS's ability to make predictions that are reasonable (i.e., not against the law of attrition by time). Among the four PSSs we tested for MC, namely SORG‐NG, SORG‐MLA, SPRING‐NG, and PATHFx, the first three had almost perfect MC. PATHFx, however, sometimes demonstrated less satisfactory MC: The MC was 0.86 when the 6‐month predictions were compared with the 12‐month predictions; and was 0.88 when the 12‐month predictions were compared with the 18‐month predictions. In the literature, none of the previous PSSs validation studies discussed model MC. Therefore, comparison could not be made to determine whether our results on these PSSs’ MCs worse than in other studies. In practice, physicians might be baffled by inconsistent predictions made by a PSS and be hesitant to use it to inform decision making. We argue that MC is an important performance metric and future PSS studies should consider reporting MCs to allow for better assessment of their models’ clinical utility.

Although survival estimation should be integrated into the decision‐making process when physicians develop the therapeutic strategy for a patient with skeletal metastasis, it is certainly not prudent to base the treatment decision solely on how long a patient could potentially live. This being said, we found in the literature only PSSs for survival estimation. No PSSs have been developed to predict other postoperative outcomes such as complications, length of hospital stay, non‐home discharge, reoperations, or quality of life.^57^, ^58^, ^59^, ^60^, ^61^ We believe all these aspects should also be discussed with patients with limited survival, who might consider quality of life as the most important goal of treatment. It would be helpful if future researchers could develop models that predict important outcomes other than survival because they might help patients and their treating physicians make better informed decisions.

## CONCLUSIONS

PSSs developed in western countries often suffer declines in performance when applied onto an external population such as our Taiwanese patients. In this study, SORG‐MLA had the best discrimination, overall performance, and provided most net clinical benefit on DCA among the eight tested PSSs. Clinicians might want to validate a specific PSS using data from their particular patient population before adopting it into practice. In the future, researchers should also consider routinely reporting important metrics such as discrimination, calibration, DCA, and MC so the models’ performance and consistency can be better compared, thus helping clinicians decide which PSS(s) may be especially suited in their clinical setting for survival prognostication and decision‐making. Furthermore, as advances in cancer treatment will impact patient survival, researchers looking to devise a new model or to refine an existing one should consider using data gathered from more up‐to‐date patients in order to maximize the performance of their predictive algorithms.

## AUTHOR CONTRIBUTIONS

**Tse‐Ying Lee:** Conceptualization (equal); formal analysis (equal); investigation (equal); methodology (equal); project administration (equal); resources (equal); validation (equal); visualization (equal); writing – original draft (equal). **Yu‐An Chen:** Data curation (equal); formal analysis (equal); project administration (equal); software (equal); visualization (equal); writing – original draft (equal). **Olivier Q. Groot:** Conceptualization (equal); data curation (equal); investigation (equal); methodology (equal); validation (equal); writing – original draft (equal); writing – review and editing (equal). **Hung‐Kuan Yen:** Conceptualization (equal); data curation (equal); formal analysis (equal); investigation (equal); methodology (equal); resources (equal); software (equal); writing – original draft (equal). **Bas Josse Jan Bindels:** Data curation (equal); formal analysis (equal); supervision (equal); validation (equal); writing – original draft (equal); writing – review and editing (equal). **Robert‐Jan Pierik:** Data curation (equal); formal analysis (equal); investigation (equal); resources (equal); supervision (equal); validation (equal); writing – original draft (equal); writing – review and editing (equal). **Hsiang‐Chieh Hsieh:** Data curation (equal); formal analysis (equal); supervision (equal); writing – review and editing (equal). **Aditya V. Karhade:** Conceptualization (equal); methodology (equal); supervision (equal); validation (equal); writing – review and editing (equal). **Ting‐En Tseng:** Conceptualization (equal); methodology (equal); visualization (equal); writing – original draft (equal). **Yi‐Hsiang Lai:** Conceptualization (equal); project administration (supporting); writing – original draft (equal). **Jiun‐Jen Yang:** Data curation (equal); formal analysis (equal); visualization (supporting); writing – original draft (supporting). **Chia‐Che Lee:** Conceptualization (equal); formal analysis (equal); supervision (equal); validation (equal); writing – review and editing (equal). **Ming‐Hsiao Hu:** Conceptualization (equal); supervision (equal); writing – review and editing (equal). **Jorrit‐Jan Verlaan:** Conceptualization (equal); methodology (equal); supervision (equal); writing – review and editing (equal). **Joseph H. Schwab:** Conceptualization (equal); methodology (equal); supervision (equal); writing – review and editing (equal). **Rong‐Sen Yang:** Conceptualization (equal); methodology (equal); validation (equal); writing – review and editing (equal). **Wei‐Hsin Lin:** Conceptualization (equal); supervision (equal); writing – review and editing (equal).

## FUNDING INFORMATION

This study was funded by the institutional project of National Taiwan University Hospital (No. 111‐N0070).

## CONFLICT OF INTEREST STATEMENT

Each author certifies that there are no commercial associations (consultancies, stock ownership, equity interest, patent/licensing arrangements, etc.) that might pose a conflict of interest in connection with the submitted article related to the author or any immediate family members.

## Supporting information


Figure S1.

Figure S2.

Figure S3.

Figure S4.

Table S1.

Table S2.

Table S3.

Table S4.

Table S5.

## Data Availability

The data that support the findings of this study are available on request from the corresponding author. The data are not publicly available due to privacy or ethical restrictions.
