# Supplementary figures and images for: Comparison of eight modern preoperative scoring systems for survival prediction in patients with extremity metastasis
Source: Cancer Med. 2023 Jun 12;12(13):14264–81. doi: 10.1002/cam4.6097 (PMC10358267; doi:10.1002/cam4.6097)

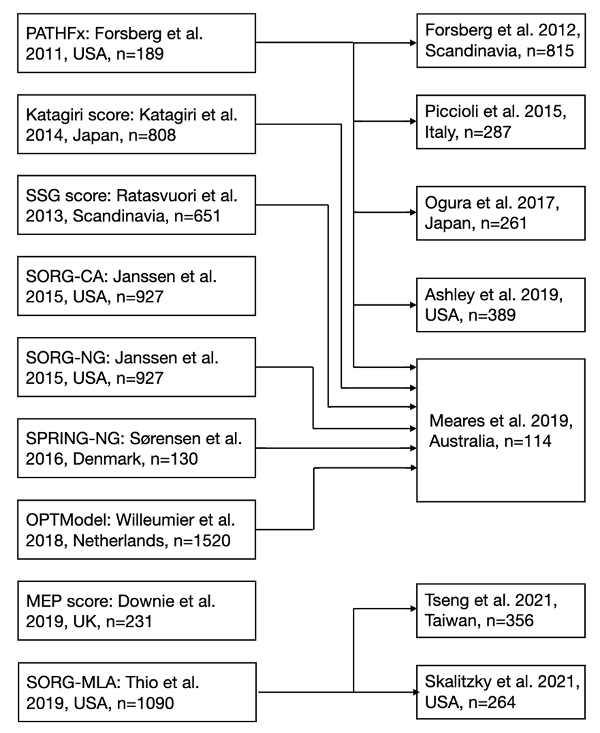

Supplement: Supplementary file 1 — Figure S1. [file CAM4-12-14264-s004.tiff]

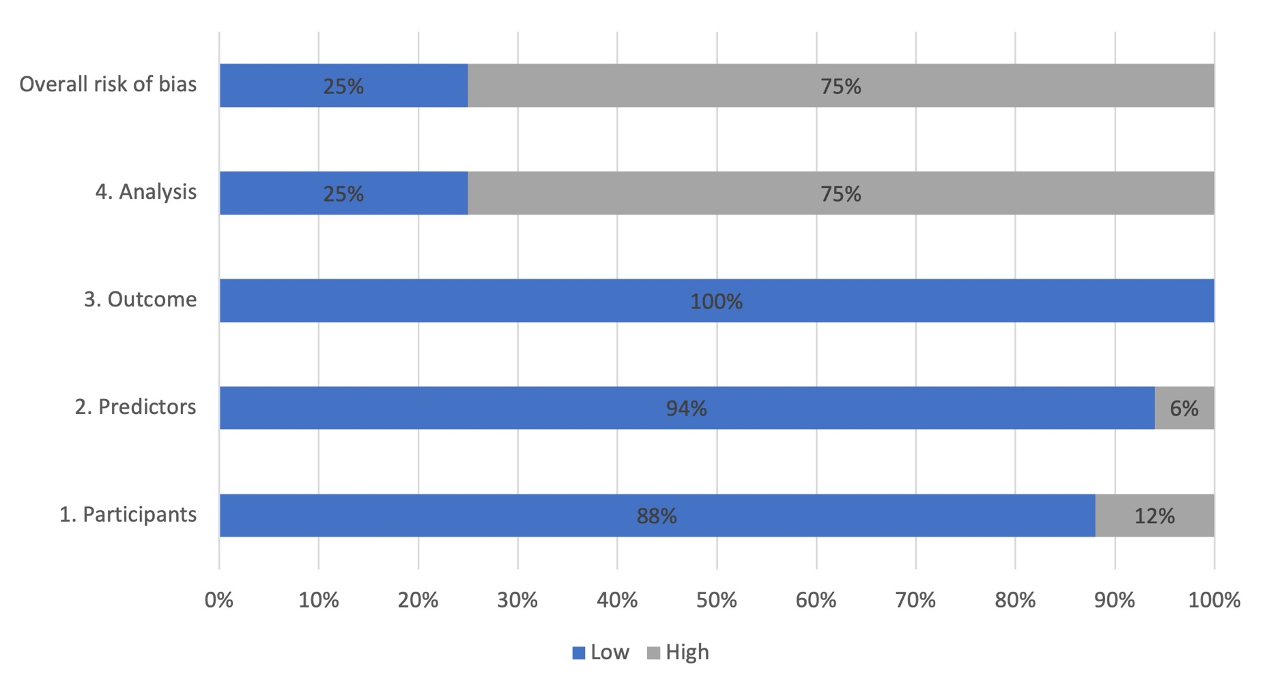

Supplement: Supplementary file 2 — Figure S2. [file CAM4-12-14264-s007.tiff]

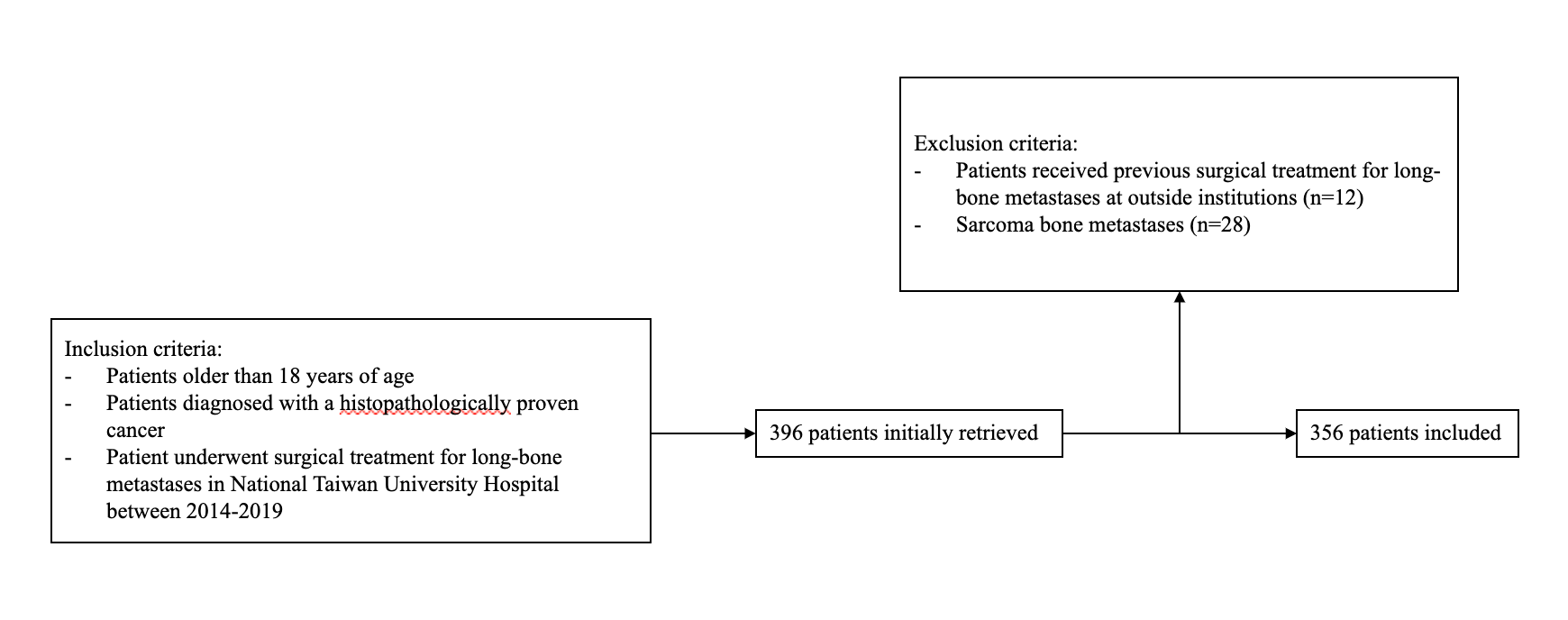

Supplement: Supplementary file 3 — Figure S3. [file CAM4-12-14264-s009.tiff]

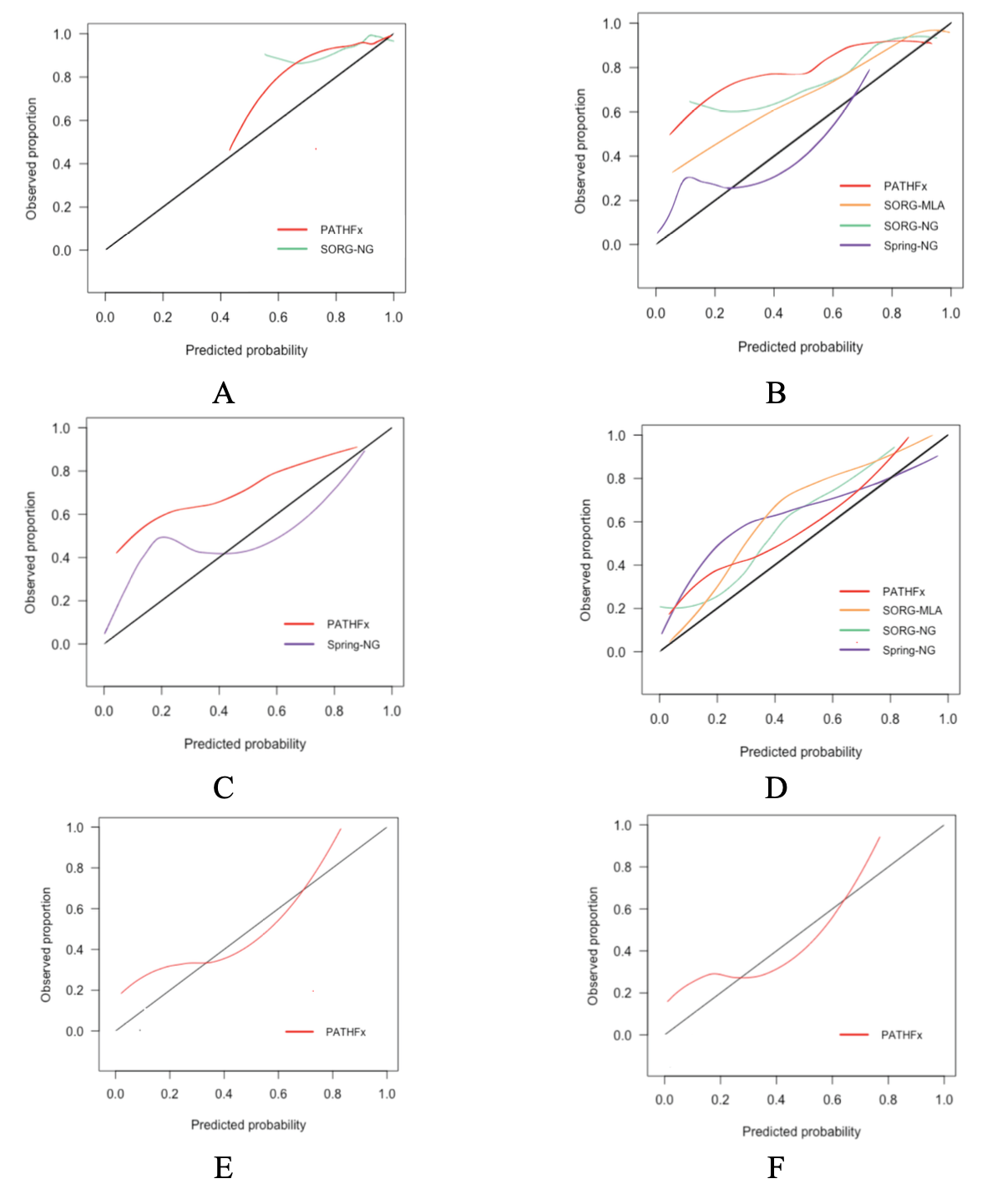

Supplement: Supplementary file 4 — Figure S4. [file CAM4-12-14264-s003.tiff]
