# Supplementary material for: Comparison of eight modern preoperative scoring systems for survival prediction in patients with extremity metastasis
Source: Cancer Med. 2023 Jun 12;12(13):14264–81. doi: 10.1002/cam4.6097 (PMC10358267; doi:10.1002/cam4.6097)
Supplement: Supplementary file 5 — Table S1. [file CAM4-12-14264-s001.docx]

| **Supplementary Table 1.**Search syntaxes for PubMed, Embase and Cochrane. |
| --- |
| ***Pubmed – December 20^th^, 2021 – 188 hits*** |
| (survival estimation OR survival prediction) AND (machine learning algorithm OR nomogram OR scoring system OR prognostic model OR prognostic system OR prediction model) AND (long-bone metastasis OR extremity metastatic disease OR extremity metastasis OR femoral metastatic bone disease OR bone metastasis of extremity) |
| ***Embase – December 20^th^, 2021 – 66 hits*** |
| (survival estimation OR survival prediction) AND (machine learning algorithm OR nomogram OR scoring system OR prognostic model OR prognostic system OR prediction model) AND (long-bone metastasis OR extremity metastatic disease OR extremity metastasis OR femoral metastatic bone disease OR bone metastasis of extremity) |
| ***Cochrane library – December 20^th^, 2021 – 1 hits*** |
| (survival estimation OR survival prediction) AND (machine learning algorithm OR nomogram OR scoring system OR prognostic model OR prognostic system OR prediction model) AND (long-bone metastasis OR extremity metastatic disease OR extremity metastasis OR femoral metastatic bone disease OR bone metastasis of extremity) |
