# Supplementary material for: Comparison of eight modern preoperative scoring systems for survival prediction in patients with extremity metastasis
Source: Cancer Med. 2023 Jun 12;12(13):14264–81. doi: 10.1002/cam4.6097 (PMC10358267; doi:10.1002/cam4.6097)
Supplement: Supplementary file 6 — Table S2. [file CAM4-12-14264-s006.docx]

Supplementary Table 2 – Risk of bias on the four major domains in PROBAST by studies

|  | Participants | Predictors | Outcome | Analysis | Overall |
| --- | --- | --- | --- | --- | --- |
| Meares^1^ | Low | Low | Low | High | High |
| Thio^2^ | Low | Low | Low | Low | Low |
| Sørensen^3^ | Low | Low | Low | High | High |
| Ratasvuori^4^ | Low | Low | Low | High | High |
| Sørensen^5^ | Low | High | Low | High | High |
| Forsberg^6^ | Low | Low | Low | High | High |
| Willeumier^7^ | Low | Low | Low | Low | Low |
| Janssen^8^ | Low | Low | Low | High | High |
| Forsberg^9^ | Low | Low | Low | Low | Low |
| Piccioli^10^ | Low | Low | Low | High | High |
| Ogura^11^ | Low | Low | Low | High | High |
| Anderson^12^ | Low | Low | Low | High | High |
| Downie^13^ | Low | Low | Low | High | High |
| Katagiri^14^ | Low | Low | High | High | High |

1. Sorensen MS, Gerds TA, Hindso K, Petersen MM. Prediction of survival after surgery due to skeletal metastases in the extremities. Bone Joint J. 2016;98-B(2):271-7.

2. Thio Q, Karhade AV, Bindels BJJ, Ogink PT, Bramer JAM, Ferrone ML, et al. Development and Internal Validation of Machine Learning Algorithms for Preoperative Survival Prediction of Extremity Metastatic Disease. Clin Orthop Relat Res. 2020;478(2):322-33.

3. Sorensen MS, Gerds TA, Hindso K, Petersen MM. Prediction of survival after surgery due to skeletal metastases in the extremities. Bone Joint J. 2016;98-B(2):271-7.

4. Ratasvuori M, Wedin R, Keller J, Nottrott M, Zaikova O, Bergh P, et al. Insight opinion to surgically treated metastatic bone disease: Scandinavian Sarcoma Group Skeletal Metastasis Registry report of 1195 operated skeletal metastasis. Surg Oncol. 2013;22(2):132-8.

5. Sorensen MS, Gerds TA, Hindso K, Petersen MM. External Validation and Optimization of the SPRING Model for Prediction of Survival After Surgical Treatment of Bone Metastases of the Extremities. Clin Orthop Relat Res. 2018;476(8):1591-9.

6. Forsberg JA, Eberhardt J, Boland PJ, Wedin R, Healey JH. Estimating survival in patients with operable skeletal metastases: an application of a bayesian belief network. PloS one. 2011;6(5):e19956.

7. Willeumier JJ, van der Linden YM, van der Wal C, Jutte PC, van der Velden JM, Smolle MA, et al. An Easy-to-Use Prognostic Model for Survival Estimation for Patients with Symptomatic Long Bone Metastases. J Bone Joint Surg Am. 2018;100(3):196-204.

8. Janssen SJ, van der Heijden AS, van Dijke M, Ready JE, Raskin KA, Ferrone ML, et al. 2015 Marshall Urist Young Investigator Award: Prognostication in Patients With Long Bone Metastases: Does a Boosting Algorithm Improve Survival Estimates? Clin Orthop Relat Res. 2015;473(10):3112-21.

9. Forsberg JA, Wedin R, Bauer HC, Hansen BH, Laitinen M, Trovik CS, et al. External validation of the Bayesian Estimated Tools for Survival (BETS) models in patients with surgically treated skeletal metastases. BMC cancer. 2012;12(1):1-8.

10. Piccioli A, Spinelli MS, Forsberg JA, Wedin R, Healey JH, Ippolito V, et al. How do we estimate survival? External validation of a tool for survival estimation in patients with metastatic bone disease—decision analysis and comparison of three international patient populations. BMC cancer. 2015;15(1):1-8.

11. Ogura K, Gokita T, Shinoda Y, Kawano H, Takagi T, Ae K, et al. Can a multivariate model for survival estimation in skeletal metastases (PATHFx) be externally validated using Japanese patients? Clinical Orthopaedics and Related Research®. 2017;475(9):2263-70.

12. Anderson AB, Wedin R, Fabbri N, Boland P, Healey J, Forsberg JA. External Validation of PATHFx Version 3.0 in Patients Treated Surgically and Nonsurgically for Symptomatic Skeletal Metastases. Clin Orthop Relat Res. 2020;478(4):808-18.

13. Downie S, Lai FY, Joss J, Adamson D, Jariwala AC. The Metastatic Early Prognostic (MEP) score. Bone Joint J. 2020;102-B(1):72-81.

14. Katagiri H, Okada R, Takagi T, Takahashi M, Murata H, Harada H, et al. New prognostic factors and scoring system for patients with skeletal metastasis. Cancer Med. 2014;3(5):1359-67.
