# Supplementary material for: Comparison of eight modern preoperative scoring systems for survival prediction in patients with extremity metastasis
Source: Cancer Med. 2023 Jun 12;12(13):14264–81. doi: 10.1002/cam4.6097 (PMC10358267; doi:10.1002/cam4.6097)
Supplement: Supplementary file 7 — Table S3. [file CAM4-12-14264-s005.docx]

| **Supplementary Table 3**. Clinical and demographic data of the included patients in the Taiwanese external validation cohort (n=356). | |
| --- | --- |
| ***Variables*** | ***% (n) \| median (range)*** |
| Demographics  Age (years)  Female sex  Body mass index (kg/m^2^)  Other Charlson comorbidities  Proportion of Hanzu | 61 (25-95)  52% (184)  23 (13-39)  60% (215)  98%^a^ |
| ECOG  0-2  3-4 | 79% (283)  21% (73) |
| ASA |  |
| 1 | 1 (1%) |
| 2 | 80 (22%) |
| 3  4 | 255 (71%)  20 (6%) |
| Primary tumor histology  Slow growth  Moderate growth  Rapid growth | 27% (97)  33% (118)  40% (141) |
| Primary tumor by location  Lung  Breast  Hepatocellular carcinoma  Renal  Prostate  Myeloma  Head and neck  Colon  Other urological  Esophageal  Thyroid  Other  Unknown  Pancreas  Gallbladder  Lymphoma  Stomach  Other gynecological  Cervical  Melanoma | 33% (116)  16% (58)  10% (36)  6% (21)  5% (19)  5% (18)  4% (16)  3% (10)  3% (9)  2% (7)  2% (6)  2% (6)  2% (6)  2% (6)  2% (6)  1% (5)  1% (4)  1% (3)  1% (2)  1% (2) |
| Pathologic fracture | 55% (195) |
| Tumor location  Upper extremity  Lower extremity | 24% (87)  76% (269) |
| Other metastases  Bone metastases  Visceral metastases (lung and/or liver)  Brain metastases | 72% (256)  51% (180)  17% (60) |
| Previous therapy  Systemic therapy  Local radiation to surgery site | 79% (281)  60% (214) |
| Preoperative laboratory values ^b^  Hemoglobin level (g/dL)  White blood cell count (10^3^/uL)  Platelet count (10^3^/uL)  Abs lymphocyte count (10^3^/uL) ^b^  Abs neutrophil count (10^3^/uL) ^b^  Neutrophil-to-lymphocyte ratio  Platelet-to-lymphocyte ratio  Albumin level (g/dL) ^b^  ALP level (IU/L) ^b^  Calcium (mg/dL) ^b^  Creatinine (mg/dL)  Sodium (mg/dL) ^b^  BUN (mg/dL) ^b^ | 11 (6-18)  7 (1-90)  234 (36-651)  1 (1-8)  5 (1-77)  5 (1-67)  216 (14-2776)  4 (1-5)  98 (23-2531)  9 (4-18)  0.7 (0.3-8.1)  137 (118-149)  16 (5.6-80) |
| *^a^Others or not disclosed by the patient: 2%*  *^b^Missing data included: absolute lymphocyte in 8 (2%) patients; absolute neutrophil count in 8 (2%) patients, albumin level in 25 (7%) patients; alkaline phosphatase level in 18 (5%) patients; calcium in 8 (2%) patients; sodium level in 1 (1%) patient; and BUN level in 89 (25%) patients.*  *Abbreviations: ECOG, Eastern Cooperative Oncology Group; ASA, American Society of Anesthesiologists; Abs, Absolute; ALP, alkaline phosphatase; BUN, Blood Urea Nitrogen* | |
