# Supplementary material for: Comparison of eight modern preoperative scoring systems for survival prediction in patients with extremity metastasis
Source: Cancer Med. 2023 Jun 12;12(13):14264–81. doi: 10.1002/cam4.6097 (PMC10358267; doi:10.1002/cam4.6097)
Supplement: Supplementary file 8 — Table S4. [file CAM4-12-14264-s008.docx]

| **Supplementary Table 4.** Sensitivity analysis showing the C-indexes and Brier scores of the eight PSSs at different time points in a subset of Taiwanese patients without missing data(n=245). | | | | | | | | |
| --- | --- | --- | --- | --- | --- | --- | --- | --- |
| **Performance metrics** | **PATHFx** | **SORG-MLA** | **SORG-CA** | **SORG-NG** | **OPTIModel** | **MEP score** | **SPRING-NG** | **SSG score** |
| C-indexes |  |  |  |  |  |  |  |  |
| 1-month | 0.69  (0.54-0.84) | - | 0.72  (0.62-0.83) | 0.77  (0.67-0.87) | 0.73  (0.58-0.88) | 0.61  (0.45-0.76) | - | 0.69  (0.54-0.83) |
| 3-months | 0.67  (0.58-0.75) | 0.82  (0.76-0.88) | 0.65  (0.57-0.72) | 0.71  (0.63-0.79) | 0.69  (0.61-0.77) | 0.67  (0.58-0.75) | 0.71  (0.63-0.79) | 0.65  (0.57-0.73) |
| 6-months | 0.62  (0.54-0.69) | - | - | - | 0.64  (0.57-0.71) | - | 0.71  (0.65-0.78) | 0.59  (0.53-0.66) |
| 12-months | 0.67  (0.60-0.74) | 0.84  (0.79-0.89) | 0.67  (0.61-0.74) | 0.76  (0.69-0.82) | 0.69  (0.62-0.75) | 0.60  (0.53-0.66) | 0.75  (0.69-0.81) | 0.64  (0.58-0.70) |
| 18-months | 0.67  (0.59-0.75) | - | - | - | 0.67  (0.60-0.74) | - | - | 0.65  (0.58-0.72) |
| 24-months | 0.64  (0.55-0.73) | - | - | - | 0.64  (0.56-0.73) | - | - | 0.62  (0.55-0.70) |
| Brier score* |  |  |  |  |  |  |  |  |
| 1-month | 0.03 (0.04) | - | 0.03 (0.04) | 0.04 (0.04) | 0.03 (0.04) | 0.04 (0.04) | - | 0.03 (0.04) |
| 3-months | 0.15 (0.15) | 0.12 (0.15) | 0.15 (0.15) | 0.14 (0.15) | 0.14 (0.15) | 0.15 (0.15) | 0.14 (0.15) | 0.15 (0.15) |
| 6-months | 0.22 (0.22) | - | - | - | 0.21 (0.22) | - | 0.20 (0.22) | 0.22 (0.22) |
| 12-months | 0.22 (0.25) | 0.16 (0.25) | 0.22 (0.25) | 0.20 (0.25) | 0.22 (0.25) | 0.24 (0.25) | 0.20 (0.25) | 0.23 (0.25) |
| 18-months | 0.20 (0.23) | - | - | - | 0.20 (0.23) | - | - | 0.21 (0.23) |
| 24-months | 0.18 (0.20) | - | - | - | 0.18 (0.20) | - | - | 0.19 (0.20) |
| *Abbreviations: C-indexes, concordance index; PSS, preoperative scoring system; SORG, Skeletal Oncology Research Group; SORG-MLA, SORG machine learning algorithm; SORG-CA, SORG classical algorithm; SORG-NG, SORG nomogram; MEP, metastatic early prognostic score; SPRING-NG, SPRING nomogram; and SSG, Scandinavian Sarcoma Group.*  **The null-model Brier score is displayed in parentheses.* | | | | | | | | |
