# Supplementary material for: Comparison of eight modern preoperative scoring systems for survival prediction in patients with extremity metastasis
Source: Cancer Med. 2023 Jun 12;12(13):14264–81. doi: 10.1002/cam4.6097 (PMC10358267; doi:10.1002/cam4.6097)
Supplement: Supplementary file 9 — Table S5. [file CAM4-12-14264-s002.docx]

| **Supplementary Table 5.** Model consistency of SORG-NG, SORG-MLA, SPRING-NG, and PATHFx. | | | | | | |
| --- | --- | --- | --- | --- | --- | --- |
|  | 1-month | 3-months | 6-months | 12-months | 18-months | 24-months |
| SORG-NG |  |  |  |  |  |  |
| 1-month | - | 1 | - | 1 | - | - |
| 3-months | 1 | - | - | 1 | - | - |
| 12-months | 1 | 1 | - | - | - | - |
| SORG-MLA |  |  |  |  |  |  |
| 3-months | - | - | - | 0.99 (0.98-1.00) | - | - |
| 12-months | - | 0.99 (0.98-1.00) | - | - | - | - |
| SPRING-NG |  |  |  |  |  |  |
| 3-months | - | - | 0.98 (0.97-0.99) | 1 | - | - |
| 6-months | - | 0.98 (0.97-0.99) | - | 1 | - | - |
| 12-months | - | 1 | 1 | - | - | - |
| PATHFx |  |  |  |  |  |  |
| 1-months | - | 1 | 1 | 1 | 1 | 1 |
| 3-months | 1 | - | 0.97 (0.95-0.98) | 0.96 (0.94-0.98) | 0.97 (0.95-0.99) | 0.99 (0.98-1.00) |
| 6-months | 1 | 0.97 (0.95-0.98) | - | 0.86 (0.83-0.90) | 0.92 (0.90-0.95) | 0.98 (0.97-1.00) |
| 12-months | 1 | 0.96 (0.94-0.98) | 0.86 (0.83-0.90) | - | 0.88 (0.85-0.92) | 0.99 (0.97-1.00) |
| 18-months | 1 | 0.97 (0.95-0.99) | 0.92 (0.90-0.95) | 0.88 (0.85-0.92) | - | 1 |
| 24-months | 1 | 0.99 (0.98-1.00) | 0.98 (0.97-1.00) | 0.99 (0.97-1.00) | 1 | - |
| *Abbreviation: SORG-NG, Skeletal Oncology Research Group nomogram; SPRING-NG, SPRING nomogram.* | | | | | | |
